# Predicting Alzheimer’s disease progression using multi-modal deep learning approach

**DOI:** 10.1038/s41598-018-37769-z

**Published:** 2019-02-13

**Authors:** Garam Lee, Kwangsik Nho, Byungkon Kang, Kyung-Ah Sohn, Dokyoon Kim, for Alzheimer’s Disease Neuroimaging Initiative, Michael W. Weiner, Paul Aisen, Ronald Petersen, Clifford R. Jack, William Jagust, John Q. Trojanowki, Arthur W. Toga, Laurel Beckett, Robert C. Green, Andrew J. Saykin, John Morris, Leslie M. Shaw, Zaven Khachaturian, Greg Sorensen, Maria Carrillo, Lew Kuller, Marc Raichle, Steven Paul, Peter Davies, Howard Fillit, Franz Hefti, Davie Holtzman, M. Marcel Mesulam, William Potter, Peter Snyder, Tom Montine, Ronald G. Thomas, Michael Donohue, Sarah Walter, Tamie Sather, Gus Jiminez, Archana B. Balasubramanian, Jennifer Mason, Iris Sim, Danielle Harvey, Matthew Bernstein, Nick Fox, Paul Thompson, Norbert Schuff, Charles DeCArli, Bret Borowski, Jeff Gunter, Matt Senjem, Prashanthi Vemuri, David Jones, Kejal Kantarci, Chad Ward, Robert A. Koeppe, Norm Foster, Eric M. Reiman, Kewei Chen, Chet Mathis, Susan Landau, Nigel J. Cairns, Erin Householder, Lisa Taylor-Reinwald, Virginia Lee, Magdalena Korecka, Michal Figurski, Karen Crawford, Scott Neu, Tatiana M. Foroud, Steven Potkin, Li Shen, Kelley Faber, Sungeun Kim, Lean Tha, Richard Frank, John Hsiao, Jeffrey Kaye, Joseph Quinn, Lisa Silbert, Betty Lind, Raina Carter, Sara Dolen, Beau Ances, Maria Carroll, Mary L. Creech, Erin Franklin, Mark A. Mintun, Stacy Schneider, Angela Oliver, Lon S. Schneider, Sonia Pawluczyk, Mauricio Beccera, Liberty Teodoro, Bryan M. Spann, James Brewer, Helen Vanderswag, Adam Fleisher, Daniel Marson, Randall Griffith, David Clark, David Geldmacher, John Brockington, Erik Roberson, Marissa Natelson Love, Judith L. Heidebrink, Joanne L. Lord, Sara S. Mason, Colleen S. Albers, David Knopman, Kris Johnson, Hillel Grossman, Effie Mitsis, Raj C. Shah, Leyla deToledo-Morrell, Rachelle S. Doody, Javier Villanueva-Meyer, Munir Chowdhury, Susan Rountree, Mimi Dang, Ranjan Duara, Daniel Varon, Maria T. Greig, Peggy Roberts, Yaakov Stern, Lawrence S. Honig, Karen L. Bell, Marilyn Albert, Chiadi Onyike, Daniel D’Agostino, Stephanie Kielb, James E. Galvin, Brittany Cerbone, Christina A. Michel, Dana M. Pogorelec, Henry Rusinek, Mony J. de Leon, Lidia Glodzik, Susan De Santi, Kyle Womack, Dana Mathews, Mary Quiceno, P. Murali Doraiswamy, Jeffrey R. Petrella, Salvador Borges-Neto, Terence Z. Wong, Edward Coleman, Allan I. Levey, James J. Lah, Janet S. Cella, Jeffrey M. Burns, Russell H. Swerdlow, William M. Brooks, Steven E. Arnold, Jason H. Karlawish, David Wolk, Christopher M. Clark, Liana Apostolova, Kathleen Tingus, Ellen Woo, Daniel H. S. Silverman, Po H. Lu, George Bartzokis, Charles D. Smith, Greg Jicha, Peter Hardy, Partha Sinha, Elizabeth Oates, Gary Conrad, Neill R. Graff-Radford, Francine Parfitt, Tracy Kendall, Heather Johnson, Oscar L. Lopez, MaryAnn Oakley, Donna M. Simpson, Martin R. Farlow, Ann Marie Hake, Brandy R. Matthews, Jared R. Brosch, Scott Herring, Cynthia Hunt, Anton P. Porsteinsson, Bonnie S. Goldstein, Kim Martin, Kelly M. Makino, M. Saleem Ismail, Connie Brand, Ruth A. Mulnard, Gaby Thai, Catherine Mc-Adams-Ortiz, Christopher H. van Dyck, Richard E. Carson, Martha G. MacAvoy, Pradeep Varma, Howard Chertkow, Howard Bergman, Chris Hosein, Sandra Black, Bojana Stefanovic, Curtis Caldwell, Ging-Yuek Robin Hsiung, Howard Feldman, Benita Mudge, Michele Assaly, Elizabeth Finger, Stephen Pasternack, Irina Rachisky, Dick Trost, Andrew Kertesz, Charles Bernick, Donna Munic, Kristine Lipowski, Masandra Weintraub, Borna Bonakdarpour, Diana Kerwin, Chuang-Kuo Wu, Nancy Johnson, Carl Sadowsky, Teresa Villena, Raymond Scott Turner, Kathleen Johnson, Brigid Reynolds, Reisa A. Sperling, Keith A. Johnson, Gad Marshall, Jerome Yesavage, Joy L. Taylor, Barton Lane, Allyson Rosen, Jared Tinklenberg, Marwan N. Sabbagh, Christine M. Belden, Sandra A. Jacobson, Sherye A. Sirrel, Neil Kowall, Ronald Killiany, Andrew E. Budson, Alexander Norbash, Patricia Lynn Johnson, Thomas O. Obisesan, Saba Wolday, Joanne Allard, Alan Lerner, Paula Ogrocki, Curtis Tatsuoka, Parianne Fatica, Evan Fletcher, Pauline Maillard, John Olichney, Owen Carmichael, Smita Kittur, Michael Borrie, T.-Y. Lee, Rob Bartha, Sterling Johnson, Sanjay Asthana, Cynthia M. Carlsson, Adrian Preda, Dana Nguyen, Pierre Tariot, Anna Burke, Nadira Trncic, Adam Fleisher, Stephanie Reeder, Vernice Bates, Horacio Capote, Michelle Rainka, Douglas W. Scharre, Maria Kataki, Anahita Adeli, Earl A. Zimmerman, Dzintra Celmins, Alice D. Brown, Godfrey D. Pearlson, Karen Blank, Karen Anderson, Laura A. Flashman, Marc Seltzer, Mary L. Hynes, Robert B. Santulli, Kaycee M. Sink, Leslie Gordineer, Jeff D. Williamson, Pradeep Garg, Franklin Watkins, Brian R. Ott, Henry Querfurth, Geoffrey Tremont, Stephen Salloway, Paul Malloy, Stephen Correia, Howard J. Rosen, Bruce L. Miller, David Perry, Jacobo Mintzer, Kenneth Spicer, David Bachman, Elizabether Finger, Stephen Pasternak, Irina Rachinsky, John Rogers, Dick Drost, Nunzio Pomara, Raymundo Hernando, Antero Sarrael, Susan K. Schultz, Laura L. Boles Ponto, Hyungsub Shim, Karen Ekstam Smith, Norman Relkin, Gloria Chaing, Michael Lin, Lisa Ravdin, Amanda Smith, Balebail Ashok Raj, Kristin Fargher

**Affiliations:** 1 Department of Software and Computer Engineering, Ajou University, Suwon, South Korea; 2 Biomedical & Translational Informatics Institute, Geisinger, Danville, USA; 3 Center for Computational Biology and Bioinformatics, Indiana University School of Medicine, Indianapolis, IN USA; 4 Center for Neuroimaging, Department of Radiology and Imaging Sciences, Indiana University School of Medicine, Indianapolis, IN USA; 5 The Huck Institute of the Life Sciences, Pennsylvania State University, University Park, USA; 6 UC San Francisco, San Francisco, CA 94107 USA; 7 UC San Diego, La Jolla, CA 92093 USA; 8 Mayo Clinic, Rochester, MN USA; 9 UC Berkeley, Berkeley, San Francisco, USA; 10 University of Pennsylvania, Philadelphia, PA 19104 USA; 11 USC, Los Angeles, CA 90032 USA; 12 UC Davis, Sacramento, CA USA; 13 Brigham and Women’s Hospital/Harvard Medical School, Boston, MA 02215 USA; 14 Indiana University, Bloomington, IN 47405 USA; 15 Washington University St. Louis, St. Loui, MO 63110 USA; 16 Prevent Alzheimer’s Disease 2020, Rockville, MD 20850 USA; 17 Siemens, Erlangen, Germany; 18 Alzheimer’s Association, Chicago, IL 60631 USA; 19 University of Pittsburg, Pittsburgh, PA 15213 USA; 20 Cornell University, Ithaca, NY 14853 USA; 21 Albert Einstein College of Medicine of Yeshiva University, Bronx, NY 10461 USA; 22 AD Drug Discovery Foundation, New York, NY 10019 USA; 23 Acumen Pharmaceuticals, Livermore, CA 94551 USA; 24 Northwestern University, Chicago, IL 60611 USA; 25 National Institute of Mental Health, Bethesda, MD 20892 USA; 26 Brown University, Providence, RI 02912 USA; 27 University of Washington, Seattle, WA 98195 USA; 28 University of London, London, UK; 29 UCLA, Torrance, CA 90509 USA; 30 University of Michigan, Ann Arbor, MI 48109-2800 USA; 31 University of Utah, Salt Lake City, UT 84112 USA; 32 Banner Alzheimer’s Institute, Phoenix, AZ 85006 USA; 33 UUC Irvine, Orange, CA 92868 USA; 34 Johns Hopkins University, Baltimore, MD 21205 USA; 35 Richard Frank Consulting, Consulting, USA; 36 National Institute on Aging, Baltimore, Maryland USA; 37 Oregon Health and Science University, Portland, OR 97239 USA; 38 University of Alabama, Birmingham, AL USA; 39 Mount Sinai School of Medicine, New York, NY USA; 40 Rush University Medical Center, Chicago, IL 60612 USA; 41 Baylor College of Medicine, Houston, TX USA; 42 Wien Center, Miami Beach, FL 33140 USA; 43 Columbia University Medical Center, New York, NY USA; 44 New York University, New York, NY USA; 45 University of Texas Southwestern Medical School, Galveston, TX 77555 USA; 46 Duke University Medical Center, Durham, NC USA; 47 Emory University, Atlanta, GA 30307 USA; 48 University of Kansas Medical Center, Kansas City, Kansas USA; 49 University of Kentucky, Lexington, KY USA; 50 Mayo Clinic, Jacksonville, Florida USA; 51 University of Rochester Medical Center, Rochester, NY 14642 USA; 52 Yale University School of Medicine, New Haven, CT USA; 53 McGill Univ. Montreal-Jewish General Hospital, Montreal, PQ H3A 2A7 Canada; 54 Sunnybrook Health Sciences, Toronto, ON Canada; 55 U.B.C. Clinic for AD & Related Disorders, Vancouver, BC Canada; 56 Cognitive Neurology - St. Joseph’s, London, ON Canada; 57 Cleveland Clinic Lou Ruvo Center for Brain Health, Las Vegas, NV 89106 USA; 58 Premiere Research Inst (Palm Beach Neurology), W Palm Beach, FL USA; 59 Georgetown University Medical Center, Washington, DC 20007 USA; 60 Stanford University, Stanford, CA 94305 USA; 61 Boston University, Boston, Massachusetts, USA; 62 Howard University, Washington, DC 20059 USA; 63 Case Western Reserve University, Cleveland, OH 44106 USA; 64 Neurological Care of CNY, Liverpool, NY 13088 USA; 65 St. Joseph’s Health Care, London, ON N6A 4H1 Canada; 66 Dent Neurologic Institute, Amherst, NY 14226 USA; 67 Ohio State University, Columbus, OH 43210 USA; 68 Albany Medical College, Albany, NY 12208 USA; 69 Hartford Hospital Olin Neuropsychiatry Research Center, Hartford, CT 06114 USA; 70 Dartmouth-Hitchcock Medical Center, Lebanon, NH USA; 71 Wake Forest University Health Sciences, Winston-Salem, NC USA; 72 Medical University South Carolina, Charleston, SC 29425 USA; 73 Nathan Kline Institute, Orangeburg, NY USA; 74 University of Iowa College of Medicine, Iowa City, IA 52242 USA; 75 University of South Florida: USF Health Byrd Alzheimer’s Institute, Tampa, FL 33613 USA

**Keywords:** Data integration, Machine learning

## Abstract

Alzheimer’s disease (AD) is a progressive neurodegenerative condition marked by a decline in cognitive functions with no validated disease modifying treatment. It is critical for timely treatment to detect AD in its earlier stage before clinical manifestation. Mild cognitive impairment (MCI) is an intermediate stage between cognitively normal older adults and AD. To predict conversion from MCI to probable AD, we applied a deep learning approach, multimodal recurrent neural network. We developed an integrative framework that combines not only cross-sectional neuroimaging biomarkers at baseline but also longitudinal cerebrospinal fluid (CSF) and cognitive performance biomarkers obtained from the Alzheimer’s Disease Neuroimaging Initiative cohort (ADNI). The proposed framework integrated longitudinal multi-domain data. Our results showed that 1) our prediction model for MCI conversion to AD yielded up to 75% accuracy (area under the curve (AUC) = 0.83) when using only single modality of data separately; and 2) our prediction model achieved the best performance with 81% accuracy (AUC = 0.86) when incorporating longitudinal multi-domain data. A multi-modal deep learning approach has potential to identify persons at risk of developing AD who might benefit most from a clinical trial or as a stratification approach within clinical trials.

## Introduction

Alzheimer’s disease (AD) is an irreversible, progressive neurodegenerative disorder characterized by abnormal accumulation of amyloid plaques and neurofibrillary tangles in the brain, causing problems with memory, thinking, and behavior. AD is the most common form of dementia with no validated disease modifying treatment. An estimated 5.7 million Americans are living with AD in 2018. By 2050, this number is projected to rise to nearly 14 million^1^. Current available treatments decelerate only the progression of AD and no treatment developed so far can cure a patient who is already in AD. Thus, it is of fundamental importance for timely treatment and progression delay to develop strategies for detection of AD at early stages before clinical manifestation. As a result, the concept of mild cognitive impairment (MCI) was introduced. MCI, a prodromal form of AD, is defined to describe people who have mild symptoms of brain malfunction but can still perform everyday tasks. Patients in the phase of MCI have an increased risk of progressing to dementia^1–4^. Some patients in their MCI stages are converted to AD within a limit of the time window after baseline, while some are not. It has been reported that MCI patients progress to AD at a rate of 10% to 15% per year and 80% of these MCI patients will have converted to AD after approximately six years of follow-up^5,6^. It is an ongoing topic among AD-related researches to identify biomarkers that classify patients with MCI who later progress to AD (MCI converter) from those with MCI who do not progress to AD (MCI non-converter).

Various machine learning methods have been applied to identify biomarkers for MCI conversion prediction and improve their performances. Support vector machine (SVM) is one of methods frequently used for solving classification problem. A lot of studies applied SVM for MCI conversion prediction^7–12^. A multi-task learning along with SVM was used to identify AD-relevant features, showing 73.9% accuracy, 68.6% sensitivity, and 73.6% specificity^7^. For the use of additional subjects, a domain transfer learning method to use auxiliary samples such as AD and cognitively normal older adults (CN) subjects as well as MCI subjects showed 79.4% accuracy, 84.5% sensitivity, and 72.7% specificity^8^. A linear discriminant analysis (LDA) was used based on cortex thickness data showing 63% sensitivity and 76% specificity^13^. Furthermore, the integration of multi-modality data improves the performance for MCI conversion prediction by extracting complementary AD-related biomarkers from each modality. Cerebrospinal fluid (CSF), MRI, and cognitive performance biomarkers were combined, resulting in 68.5% accuracy 53.4% sensitivity, and 77% specificity^14,15^. Along with MRI and CSF biomarkers, *APOE* ε4 status were integrated^16^.

In this study, in order to predict MCI to AD conversion, we proposed a multimodal recurrent neural network method, a deep learning approach, based on the integration of demographic information, longitudinal CSF biomarkers, longitudinal cognitive performance, and cross-sectional neuroimaging biomarkers at baseline obtained from the Alzheimer’s Disease Neuroimaging Initiative cohort (ADNI). Our proposed deep learning method can incorporate longitudinal multiple domain data and take variable-length longitudinal data to capture temporal features at multiple time points. In particular, non-overlapping samples as well as overlapping samples from each data can be used to build a prediction model.

## Results

### Study participants

All individuals used in the analysis were participants of the Alzheimer’s Disease Neuroimaging Initiative (ADNI)^17,18^. The overall goal of ADNI is to test whether serial magnetic resonance imaging (MRI), position emission tomography (PET), other biological markers, and clinical and neuropsychological assessment could be combined to measure the progression of MCI and early AD. Demographic information, raw neuroimaging scan data, *APOE* genotype, CSF measurements, neuropsychological test scores, and diagnostic information are publicly available from the ADNI data repository (http://adni.loni.usc.edu). Informed consent was obtained for all subjects, and the study was approved by the relevant institutional review board at each data acquisition site (for up-to-date information, see http://adni.loni.usc.edu/wp-content/themes/freshnews-dev-v2/documents/policy/ADNI_Acknowledgement_List%205-29-18.pdf). All methods were performed in accordance with the relevant guidelines and regulations. In this study, a total of 1,618 ADNI participants aged 55 to 91 were used, which include 415 cognitively normal older adult controls (CN), 865 MCI (307 MCI converter and 558 MCI non-converter), and 338 AD patients (Table 1).

**Table 1:** Subject demographics at baseline visit.

Characteristics	MCI-C (n = 307)	MCI-NC (n = 558)	CN (n = 415)	AD (n = 338)
Sex (Female/Male)	117/190	236/322	206/209	152 /186
Memory score (mean ± sd)	−0.26 ± 0.5	0.39 ± 0.64	1.01 ± 0.56	−0.86 ± 0.54
Education (mean ± sd)	15.89 ± 2.75	15.94 ± 2.88	16.28 ± 2.72	15.15 ± 2.99
*APOE* ε4 (0/1/2)	105/153/49	325/188/45	301/103/11	113/160/65

We used four different types, or modalities of data: demographic information, neuroimaging phenotypes measured by MRI, cognitive performance, and CSF measurements. Demographic information includes age, sex, years of education, and *APOE ε4* status. Cognitive performance includes composite scores for executive functioning (ADNI-EF) and memory (ADNI-MEM) derived from the ADNI neuropsychological battery using item response theory as described in detail elsewhere^19^. CSF biomarkers for AD include amyloid-β 1–42 peptide (Aβ_1–42_), total tau (t-tau), and tau phosphorylated at the threonine 181 (p-tau). AD-related neuroimaging biomarkers measured by MRI include hippocampal volume and entorhinal cortical thickness.

### Experimental setting

To evaluate the performance and effectiveness of our proposed longitudinal multi-modal deep learning method, we used three schemes and compared their performances (Table 2). In the experiment named “*baseline*”, 4 modalities data at baseline visit (cognitive performance, CSF, demographic information, and MRI) were incorporated. In “*single modal*”, only longitudinal cognitive performance data was used for the predictor (we tried all other single modality, and the performance with cognitive scores was the best). Finally, the four modalities of longitudinal data were combined and used for training the classifier in the experiment marked as “*proposed*”. Table 3 shows summary statistics of each modalities of data and hyperparameters used for training GRUs.

**Table 2:** Three experimental schemes depending on training dataset composition.

Longitudinal data	Multimodal data	Representation
	✓	*baseline*
✓		*single modal*
✓	✓	*proposed*

**Table 3:** Summary statistics for data and hyperparameters.

	#Features	Hidden Dimension	Time length (Average)	Time length (sd)
Cognitive performance	2	3	3.7	1.32
Demographic Information	4	5	1	0
CSF	5	6	1.4	0.5
MRI	3	4	1	0

For training our models, subjects in CN and AD groups are used as well as MCI-C and MCI-NC. This approach is motivated by^8,10,11,20–22^. They use CN and AD subjects for training a classifier such as SVM^23^ or locally linear embedding (LLE)^24^, and then the classifier is used for classification of MCI-C and MCI-NC. In our experiment, CN and AD are used as auxiliary dataset to pre-train the classifier, and then MCI-C and MCI-NC are also used for training.

**Figure 1:**
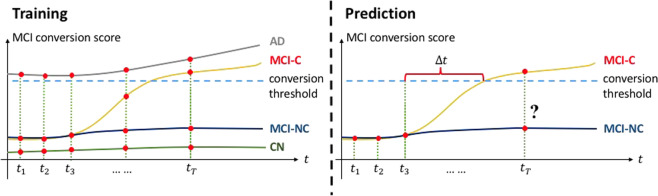
An example using longitudinal data for MCI conversion prediction. Contrary to the experiment with baseline visit data, longitudinal data of individuals in all stages (CN, MCI, and AD) was used for training a classifier. Then, portion of longitudinal data was taken to the classifier to predict AD progression after Δ*t*.

**Figure 2:**
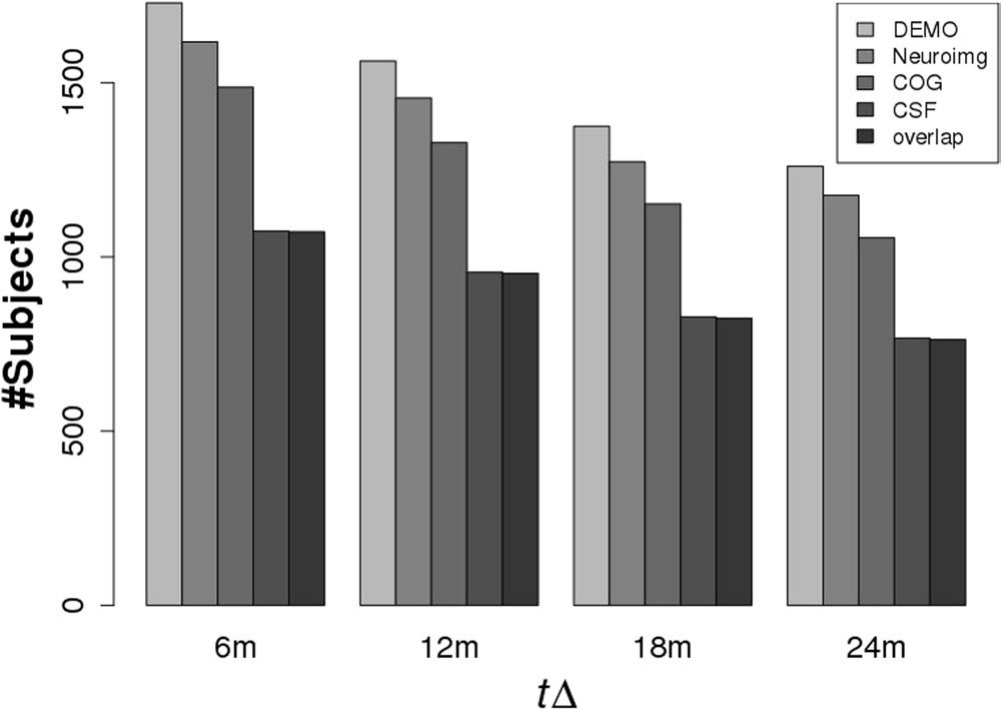
The number of subjects available in demographic data, neuroimaging data, cognitive performance, and CSF biomarkers over ∆*t*.

We tested the classifier on MCI patients to predict the conversion after Δ*t* from baseline (6, 12, 18, and 24 months) as shown in Fig. 1. Due to the nature of our data the sample size available for training varies over Δ*t* (Fig. 2). For example, if AD occurs early from the baseline visit, then we have relatively fewer training samples because we have a smaller data window to predict on. At each prediction time (Δ*t*), we ran 5-fold cross-validation 10 times in which every fold has the same ratio of MCI-C and MCI-NC subjects. MCI samples were partitioned into 5 subsets, and one subset was selected for testing, while the remaining subsets were used for training.

### Comparison of prediction of MCI to AD conversion using cross-sectional data at baseline and longitudinal data

**Figure 3:**
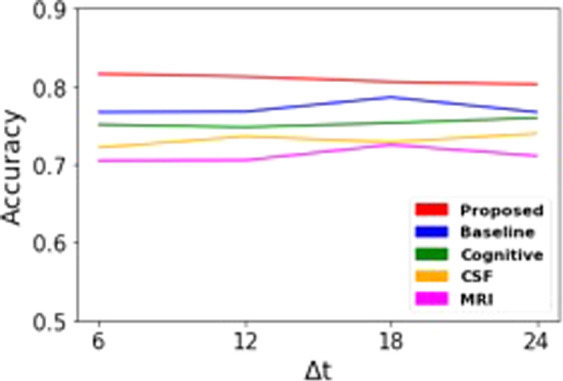
Predictive performances with “*proposed*”, “*baseline*”, and “*single modal*”. *Abbreviations*: COG: cognition performance biomarkers; CSF: cerebrospinal fluid biomarkers; NeuroImg: MRI biomarkers.

**Figure 4:**
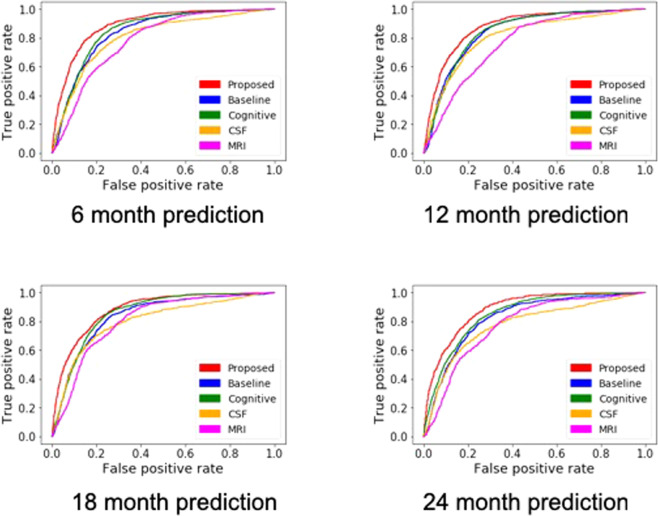
ROC curves from the “*proposed*”, “*baseline*”, and each “*single modal*” method. (**a**) 6 month prediction. (**b**) 12 month prediction. (**c**) 18 month prediction. (**d**) 24 month prediction.

To evaluate the advantage of using longitudinal data, we first compared the performances of two schemes: “*baseline*” and “*proposed*” (Figs 3 and 4). Intuitively, data from multiple time points has more information than data at a single time point. Thus, the GRU analyzes the temporal changes in cognitive performance and CSF to extract features (which are not contained in baseline visit data) for the correct MCI conversion prediction. As shown in Table 4(a,b), the prediction model based on longitudinal data shows better performance than the model using only cross-sectional data at baseline. In particular, sensitivity is an important measure for the prediction task in which identifying true positive rate is crucial^25^. In prediction of MCI conversion, a classifier with higher true positive rate is more applicable for timely treatment.

**Table 4:** Prediction performance based on Δ*t* over different schemes.

Δ*t*	6 m	12 m	18 m	24 m
(a) Multimodal longitudinal data (*proposed*)				
ACC (mean ±sd)	0.81 ± 0.03	0.81 ± 0.03	0.79 ± 0.03	0.80 ± 0.03
SEN (mean ± sd)	0.84 ± 0.07	0.84 ± 0.05	0.82 ± 0.07	0.81 ± 0.10
SPE (mean ± sd)	0.80 ± 0.04	0.80 ± 0.04	0.79 ± 0.04	0.80 ± 0.04
(b) Multimodal baseline visit data (*baseline*)				
ACC (mean ±sd)	0.76 ± 0.03	0.76 ± 0.03	0.78 ± 0.03	0.76 ± 0.03
SEN (mean ± sd)	0.81 ± 0.07	0.82 ± 0.07	0.81 ± 0.07	0.80 ± 0.08
SPE (mean ± sd)	0.80 ± 0.04	0.75 ± 0.03	0.77 ± 0.05	0.76 ± 0.03
(c) Cognitive performance data (*single modal*)				
ACC (mean ±sd)	0.74 ± 0.06	0.75 ± 0.05	0.74 ± 0.04	0.74 ± 0.04
SEN (mean ± sd)	0.81 ± 0.07	0.78 ± 0.14	0.76 ± 0.20	0.76 ± 0.20
SPE (mean ± sd)	0.75 ± 0.08	0.71 ± 0.05	0.71 ± 0.06	0.71 ± 0.06

### Comparison of prediction of MCI to AD conversion using single modal and multimodal data

For evaluating the effectiveness of multimodal data integration, we compared the performances of “*proposed*” and “*single modal*” experiments. Figure 3 shows the accuracies of “*proposed*” and models with single modality of data. We removed the accuracy from the model with demographic data because the prediction performance was too low. The model using cognitive performance was observed to be the most accurate among models that use each single modality of data. Even though the sample size for neuroimaging data was larger than those of cognitive performance and CSF biomarkers (Fig. 3), the model with neuroimaging data showed less accuracy. This is because cognitive performance is a longitudinal data which takes advantage of giving relatively closer data record to MCI conversion point. However, model with cognitive performance shows extremely high variance of sensitivity for predicting 18 and 24 months. It is observed that model only with cognitive performance not a stable predictor for long period of prediction while integrating other biomarkers can alleviate the high variance in *proposed*.

## Discussion

We proposed an integrative approach for the prediction of MCI to AD conversion using a deep learning approach, more specifically, a multi-modal recurrent neural network. Our method takes advantages of longitudinal and multi-modal nature of available data to discover nonlinear patterns associated with MCI progression. To evaluate the advantages of our proposed method, we compared performance outputs from three schemes: “*baseline*”, “*single modal*”, and “*proposed*”. As observed in Fig. 4, *“baseline”* and *“single modal”* with cognitive test biomarkers show similar performances over prediction periods. Using longitudinal data or combining multimodal data are effective ways for increasing predictive power thus, it seems natural for combining longitudinal multimodal data (*“proposed”*) to show the best performance. In Table 5, as predicted further periods, the reliability of performance improvement is lower due to the lack of positive samples. However, specificities of proposed model showed enhanced performance over competing methods consistently. In addition, the prediction results of our model were compared to those of previous studies with machine learning approaches (Table 6). Our method showed comparable prediction ability even though we had a highly unbalanced ratio of positive and negative samples. Specifically, the sensitivity of our model shows higher performance while specificity is lower. Moreover, The balanced accuracy^26^, which is a measure of accuracy considering sensitivity and specificity shows 0.82 for our model and 0.81 for^27^.

**Table 5:** Performance comparison between different models. P-values are calculated using a paired t-test between the proposed and each competing method.

Model	accuracy (p-value)	sensitivity (p-value)	specificity (p-value)
**(a) 6 month prediction**.			
Proposed	0.81	0.84	0.80
Baseline	0.76 (1.16e-08)	0.81 (0.07)	0.75 (1.87e-10)
Single modal (cognitive performance)	0.74 (3.17e-09)	0.81 (8.12e-05)	0.70 (2.68e-14)
Single modal (CSF)	0.72 (4.65e-17)	0.78 (8.12e-05)	0.70 (2.68e-14)
Single modal (MRI)	0.70 (4.68e-24)	0.75 (6.71e-10)	0.68 (2.05e-21)
**(b) 12 month prediction**			
Proposed	**0.81**	**0.84**	**0.80**
Baseline	0.76 (1.77e-07)	0.82 (0.057)	0.75 (3.65e-07)
Single modal (cognitive performance)	0.75 (2.70e-08)	0.78 (0.007)	0.71 (1.73e-09)
Single modal (CSF)	0.73 (3.47e-20)	0.78 (1.40e-05)	0.72 (3.90e-16)
Single modal (MRI)	0.70 (7.29e-22)	0.73 (1.03e-12)	0.69 (2.07e-16)
**(c) 18 month prediction**			
Proposed	**0.79**	**0.82**	**0.79**
Baseline	0.78 (0.08)	0.81 (0.64)	0.77 (0.15)
Single modal (cognitive performance)	0.74 (2.43e-06)	0.76 (0.03)	0.71 (7.72e-08)
Single modal (CSF)	0.72 (9.84e-12)	0.78 (0.07)	0.71 (6.55e-11)
Single modal (MRI)	0.72 (5.26e-14)	0.76 (0.16e-04)	0.71 (2.20e-10)
**(d) 24 month prediction**			
Proposed	**0.80**	**0.81**	**0.80**
Baseline	0.76 (2.12e-05)	0.80 (0.57)	0.76 (8.95e-05)
Single modal (cognitive performance)	0.74 (4.69e-07)	0.76 (0.15)	0.72 (1.20e-09)
Single modal (CSF)	0.73 (1.06e-09)	0.73 (0.001)	0.74 (3.16e-07)
Single modal (MRI)	0.71 (1.18e-12)	0.76 (0.03)	0.70 (2.53e-10)

**Table 6:** A list of previous models that train a classifier mainly using MCI samples.

Method	Subjects (MCI-C/MCI-NC)	Data source	ACC	SEN	SPE
SVM^7^	43/48	MRI, PET, CSF	0.73	0.68	0.73
SVM^8^	43/56	MRI, FDG-PET, CSF	0.79	0.84	0.72
SVM^9^	35/50	MRI, PET, cognitive score	0.78	0.79	0.78
Gaussian process^39^	47/96	MRI, PET, CSF, APOE genotype	0.68	**0.90**	0.52
Hierarchical ensemble^25^	70/61	MRI	0.79	0.86	0.78
Deep neural network^27^	235/409	MRI, PET	**0.82**	0.79	**0.83**
Proposed	134/561	Cognitive score, MRI, CSF biomarker, demographic data	0.81	0.84	0.80

The biggest advantage of our approach is that irregular longitudinal data can be used. One of the major problems when dealing with longitudinal data is that a preprocessing step is required for handling variable-length of sequential data and missing values. In previous studies, the fixed length of time points was collected by taking data that fell within a certain time window. Additionally, an additional feature extraction phase is required to produce a fixed-size feature representation. In the first training step, separate GRU components make an encoding process, where longitudinal data are transformed into a vector containing AD-sensitive features. Thanks to the structure of GRU, our approach is capable of accepting any irregular length of data as an input without preprocessing.

In addition, our method can make full use of available subjects from each modality for training our classifier. This is a huge advantage in the face of data scarcity. As seen in Fig. 2, the number of subjects with CSF data is smallest in the overlapping sample. Traditional approaches can use only the overlapping samples while non-overlapping samples were abandoned. In our case, non-overlapping samples contribute to training the individual GRU component it belongs to for the better representation learning. Furthermore, additional modality data are easily integrated into the model. Contrary to the kernel-based integration, concatenation-based integration method can incorporate other domains of data such as multi-modal neuroimaging and genomic data without any prior knowledge. Thus, next we will integrate multi-modal neuroimaging and genomics data for learning features that might be useful in predicting early MCI to AD conversion.

Although there are some strengths as described above, our approach has some limitations. In the first training step, the input of each modality was transformed into a feature vector that is optimized for MCI conversion prediction only by single modality. Thus, features that are irrelevant to AD progression with respect to the single modality will be filtered out. However, if there are features that cannot be extracted by single modality but only can be explained by a combination of multi-modality of data then those are also likely to be filtered out. This is because parameters in GRUs are not updated against the final prediction result. In other words, parameter optimization for the second training step does not affect the parameters in each GRU for feature extraction, thus each GRU cannot learn from the final prediction based on the combined features. To solve this problem, we will link GRUs to logistic regressions at the second step so that GRU learns feature representation from multi-modality as well as single modality. In addition, we plan to modify the structure of our model making it possible for individual GRU components to extract integrative features. We are currently investigating this possibility as a sequel to this work.

## Methods

### Recurrent Neural Network

Recurrent Neural Network (RNN) is a class of deep learning architecture used when sequential data can be considered. In natural language processing (NLP), speech recognition, and anomaly detection in time series, RNN is popularly used for analyzing the sequence of words and time series data^28^. The advantage of applying RNN is that variable-length sequence can be processed to exploit temporal patterns hidden in the given sequence. In the sentiment analysis task, for example, the goal is to classify the sentiment (good or bad) of a given sentence. The classifier needs to take a sentence (a sequence of words) as an input, understand the context in it, and return a correct sentiment as an output^29^. For the prediction task that detects initial diagnosis of heart failure in^30^, RNN takes time series of electronic health records (EHRs) using 12 to 18 month observation window. In these cases where variable-length input should be dealt with RNN is an appropriate candidate to use.

**Figure 5:**
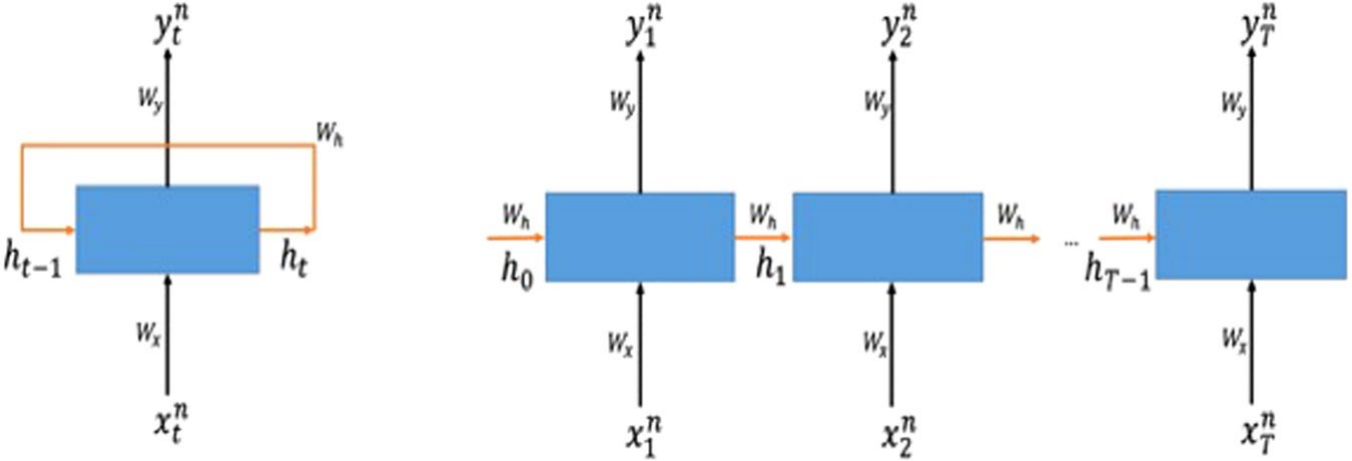
Illustration of recurrent neural network. RNN is composed of input, memory state, and output, each of which has a weight parameter to be learned for a given task. The memory state (blue box) takes the input and computes the output based on the memory state from the previous step and the current input (left). Since the RNN has a feedback loop, variable-length input and output sequence can be represented as an “unfolded” sequence (right).

An RNN processes one element of an input sequence at a time and updates its memory state that implicitly contains information about the history of all the past elements of the sequence^31^. The hidden state is represented as a Euclidean vector (i.e., a sequence of real numbers) and is updated recursively from the input at the given step and the previous value of the hidden state (Fig. 5).

Suppose we have N number of subjects, each of which has a sequence $\{{x}_{1}^{n},{x}_{2}^{n},\ldots ,{x}_{t}^{n},\ldots ,{x}_{T}^{n}\}$ where ${x}_{i}^{n}$ is a data record of *n*-th sample and the *t*-th element in a sequence and T is the length of the sequence. The corresponding sequence of output is recursively computed as:

1 $${h}_{t}=tanh({W}_{h}{h}_{t-1}+{W}_{x}{x}_{t}^{n})$$

2 $${\hat{y}}_{t}=\sigma ({W}_{y}{h}_{t})$$

*W*_*h*_, *W*_*x*_, and *W*_*y*_ are the weight matrices to extract task-specific features from the previous memory state *h*_*t*−1_, the *t*-th input *x*_*t*_ and the current memory state *h*_*t*_, respectively. As can be seen from the equations, the memory states h_t_ and the input x_t_ are all represented as Euclidean vectors as well. Therefore, the dynamics of the entire RNN are captured by a sequence of matrix-vector multiplications, followed by elementwise non-linearity applications. The *tanh* function is a non-linear activation function taking the form of $tanh(x)=\frac{2}{1+{e}^{-2x}}-1$. The role of the non-linear activation function is to endow the RNN with higher representational power. ${\hat{y}}_{t}$ is the predicted output resulting from the computation of the network. This final result is computed by the function σ, which is known as the softmax function. The role of the softmax function is to turn an arbitrary vector into a probability vector via the following operation:

$$\sigma ({u}_{i})=\frac{{e}^{{u}_{i}}}{{\sum }_{k}{e}^{{u}_{k}}}$$

where *u*_*i*_ is the i-th element of the vector *u*. In equation (2), we abuse notation to express elementwise application of the above expression.

3 $$L(y,{\hat{y}}_{T})=\frac{1}{N}\sum _{n\in N}{y}_{n}log{\hat{y}}_{T}^{n}$$

4 $${W}_{h}^{\ast },{W}_{x}^{\ast },{W}_{y}^{\ast }=\mathop{argmin}\limits_{{W}_{h},{W}_{x},{W}_{y}}\,L(y,{\hat{y}}_{T})$$

In our model, the last output sequence provided by the RNN is treated as the probability vector for classification, and the cross-entropy loss function (equation (3)) is used to quantify how “far away” our n-th prediction is from the n-th ground truth label y_n_. That is, we choose the optimal parameters ${{\rm{W}}}_{{\rm{h}}}^{\ast },{{\rm{W}}}_{{\rm{x}}}^{\ast },{{\rm{W}}}_{{\rm{y}}}^{\ast }$ that minimize the cross-entropy loss of the given data (equation (4)). The algorithm we use to optimize the parameters is Backpropagation Through Time (BPTT)^32^, which updates the weights in the RNN to minimize the given loss function.

However, when the task requires long sequences of input to be processed, training an RNN is difficult^33^. This is called the long-term dependency problem. Variants of RNN such as Long Short-Term Memory (LSTM) and Gated Recurrent Unit (GRU) have been developed and practically used to solve this problem^34,35^. In the proposed model, we use GRU for each modality of data to process multiple time points of the input. The detailed structure of GRU is described in the supplementary.

### Multi-modal GRU for MCI conversion prediction

Our problem can be considered as a sequential data classification. The classification objective is to predict whether an individual with MCI at baseline is converted to AD or not using sequence data, which consist of four modalities including cognitive performance, CSF, and MRI biomarkers as well as demographic information. Even though demographic data and MRI biomarkers are not longitudinal data we will consider them as length-one sequential data.

**Figure 6:**
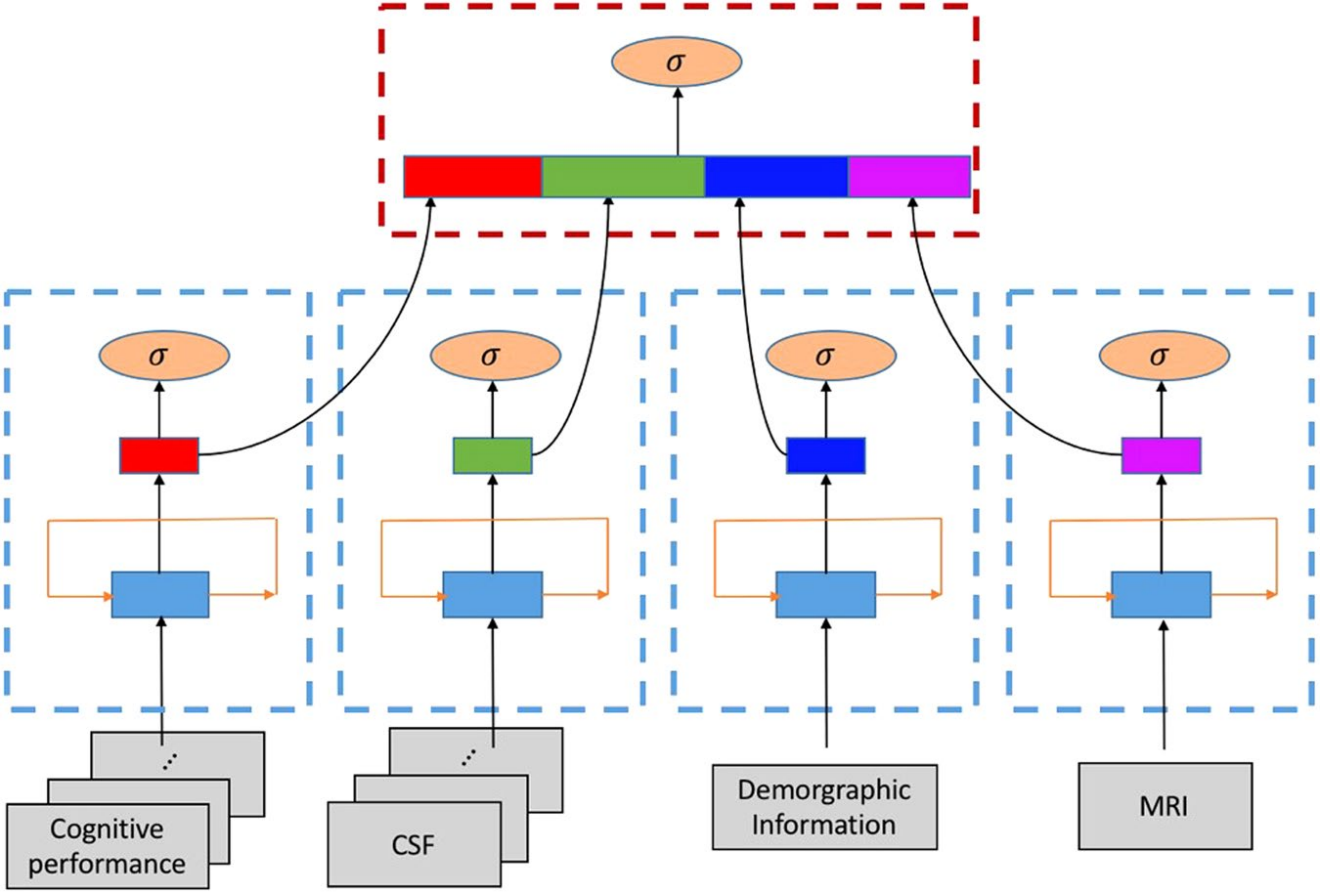
Overview of the proposed method. Our proposed method contains multiple GRU components that accept each modality of the dataset. At the first training step (blue dashed rectangle), each GRU component takes both time series or non-time series data to produce fixed-size feature vectors. And then the vectors are concatenated to form an input for the final prediction in the second training step (red dashed rectangle).

To apply a GRU-based classification algorithm to our problem, we need to design a model that can incorporate the four modalities of data. The main idea of our model is to separately build GRU feature extractors for each modality and integrate the extracted four feature vectors at the end. Our model is comprised of two training steps: (1) learning a single GRU for each modality of data, and (2) learning the integrative feature representation to make the final prediction. At the first training step, a single GRU is trained separately for each modality in which the classification objective is to predict conversion to AD from MCI. Using GRUs is essential to take longitudinal data and transform them into a fixed-size vector. This is quite similar to the approach proposed in^36^ that maps the input sequence into fixed-length representation. In the second step, MCI conversion is predicted based on the four vectors produced from each GRU components. For merging four vectors, we select concatenation-based data integration, which is conceptually the simplest method to integrate multiple sources of data into a single vector^37^. For the final prediction, *l*_1_-regularized logistic regression^38^ is used for the classification between MCI-C and MCI-NC. The overview of our proposed method is illustrated in Fig. 6.

## Conclusion

Here, we proposed a multi-modal deep learning approach to study the prediction of MCI to AD conversion using longitudinal cognitive performance and CSF biomarkers as well as cross-sectional neuroimaging and demographic data at baseline. We applied multiple GRUs to use longitudinal multi-domain data and all subjects with each modality data. Our results showed that we achieved the better prediction accuracy of MCI to AD conversion by incorporating longitudinal multi-domain data. A multi-modal deep learning approach has potential to identify persons at risk of developing AD who might benefit most from a clinical trial or as a stratification approach within clinical trials.

## Data Availability

Demogra phic information, neuroimaging data, APOE genotype, CSF measurements, neuropsychological test scores, and diagnostic information are publicly available from the ADNI data repository (http://adni.loni.usc.edu).
