# Author Correction: Predicting Alzheimer’s disease progression using multi-modal deep learning approach

**DOI:** 10.1038/s41598-023-39138-x

**Published:** 2023-08-01

**Authors:** Garam Lee, Kwangsik Nho, Byungkon Kang, Kyung-Ah Sohn, Dokyoon Kim, Alzheimer’s Disease Neuroimaging Initiative, Michael W. Weiner, Paul Aisen, Ronald Petersen, Clifford R. Jack, William Jagust, John Q. Trojanowki, Arthur W. Toga, Laurel Beckett, Robert C. Green, Andrew J. Saykin, John Morris, Leslie M. Shaw, Zaven Khachaturian, Greg Sorensen, Maria Carrillo, Lew Kuller, Marc Raichle, Steven Paul, Peter Davies, Howard Fillit, Franz Hefti, Davie Holtzman, M. Marcel Mesulam, William Potter, Peter Snyder, Tom Montine, Ronald G. Thomas, Michael Donohue, Sarah Walter, Tamie Sather, Gus Jiminez, Archana B. Balasubramanian, Jennifer Mason, Iris Sim, Danielle Harvey, Matthew Bernstein, Nick Fox, Paul Thompson, Norbert Schuff, Charles DeCArli, Bret Borowski, Jeff Gunter, Matt Senjem, Prashanthi Vemuri, David Jones, Kejal Kantarci, Chad Ward, Robert A. Koeppe, Norm Foster, Eric M. Reiman, Kewei Chen, Chet Mathis, Susan Landau, Nigel J. Cairns, Erin Householder, Lisa Taylor-Reinwald, Virginia Lee, Magdalena Korecka, Michal Figurski, Karen Crawford, Scott Neu, Tatiana M. Foroud, Steven Potkin, Li Shen, Kelley Faber, Sungeun Kim, Lean Tha, Richard Frank, John Hsiao, Jeffrey Kaye, Joseph Quinn, Lisa Silbert, Betty Lind, Raina Carter, Sara Dolen, Beau Ances, Maria Carroll, Mary L. Creech, Erin Franklin, Mark A. Mintun, Stacy Schneider, Angela Oliver, Lon S. Schneider, Sonia Pawluczyk, Mauricio Beccera, Liberty Teodoro, Bryan M. Spann, James Brewer, Helen Vanderswag, Adam Fleisher, Daniel Marson, Randall Griffith, David Clark, David Geldmacher, John Brockington, Erik Roberson, Marissa Natelson Love, Judith L. Heidebrink, Joanne L. Lord, Sara S. Mason, Colleen S. Albers, David Knopman, Kris Johnson, Hillel Grossman, Effie Mitsis, Raj C. Shah, Leyla deToledo-Morrell, Rachelle S. Doody, Javier Villanueva-Meyer, Munir Chowdhury, Susan Rountree, Mimi Dang, Ranjan Duara, Daniel Varon, Maria T. Greig, Peggy Roberts, Yaakov Stern, Lawrence S. Honig, Karen L. Bell, Marilyn Albert, Chiadi Onyike, Daniel D’Agostino, Stephanie Kielb, James E. Galvin, Brittany Cerbone, Christina A. Michel, Dana M. Pogorelec, Henry Rusinek, Mony J. de Leon, Lidia Glodzik, Susan De Santi, Kyle Womack, Dana Mathews, Mary Quiceno, P. Murali Doraiswamy, Jeffrey R. Petrella, Salvador Borges-Neto, Terence Z. Wong, Edward Coleman, Allan I. Levey, James J. Lah, Janet S. Cella, Jeffrey M. Burns, Russell H. Swerdlow, William M. Brooks, Steven E. Arnold, Jason H. Karlawish, David Wolk, Christopher M. Clark, Liana Apostolova, Kathleen Tingus, Ellen Woo, Daniel H. S. Silverman, Po H. Lu, George Bartzokis, Charles D. Smith, Greg Jicha, Peter Hardy, Partha Sinha, Elizabeth Oates, Gary Conrad, Neill R. Graff-Radford, Francine Parfitt, Tracy Kendall, Heather Johnson, Oscar L. Lopez, MaryAnn Oakley, Donna M. Simpson, Martin R. Farlow, Ann Marie Hake, Brandy R. Matthews, Jared R. Brosch, Scott Herring, Cynthia Hunt, Anton P. Porsteinsson, Bonnie S. Goldstein, Kim Martin, Kelly M. Makino, M. Saleem Ismail, Connie Brand, Ruth A. Mulnard, Gaby Thai, Catherine Mc-Adams-Ortiz, Christopher H. van Dyck, Richard E. Carson, Martha G. MacAvoy, Pradeep Varma, Howard Chertkow, Howard Bergman, Chris Hosein, Sandra Black, Bojana Stefanovic, Curtis Caldwell, Ging-Yuek Robin Hsiung, Howard Feldman, Benita Mudge, Michele Assaly, Elizabeth Finger, Stephen Pasternack, Irina Rachisky, Dick Trost, Andrew Kertesz, Charles Bernick, Donna Munic, Kristine Lipowski, Masandra Weintraub, Borna Bonakdarpour, Diana Kerwin, Chuang-Kuo Wu, Nancy Johnson, Carl Sadowsky, Teresa Villena, Raymond Scott Turner, Kathleen Johnson, Brigid Reynolds, Reisa A. Sperling, Keith A. Johnson, Gad Marshall, Jerome Yesavage, Joy L. Taylor, Barton Lane, Allyson Rosen, Jared Tinklenberg, Marwan N. Sabbagh, Christine M. Belden, Sandra A. Jacobson, Sherye A. Sirrel, Neil Kowall, Ronald Killiany, Andrew E. Budson, Alexander Norbash, Patricia Lynn Johnson, Thomas O. Obisesan, Saba Wolday, Joanne Allard, Alan Lerner, Paula Ogrocki, Curtis Tatsuoka, Parianne Fatica, Evan Fletcher, Pauline Maillard, John Olichney, Owen Carmichael, Smita Kittur, Michael Borrie, T.-Y. Lee, Rob Bartha, Sterling Johnson, Sanjay Asthana, Cynthia M. Carlsson, Adrian Preda, Dana Nguyen, Pierre Tariot, Anna Burke, Nadira Trncic, Adam Fleisher, Stephanie Reeder, Vernice Bates, Horacio Capote, Michelle Rainka, Douglas W. Scharre, Maria Kataki, Anahita Adeli, Earl A. Zimmerman, Dzintra Celmins, Alice D. Brown, Godfrey D. Pearlson, Karen Blank, Karen Anderson, Laura A. Flashman, Marc Seltzer, Mary L. Hynes, Robert B. Santulli, Kaycee M. Sink, Leslie Gordineer, Jeff D. Williamson, Pradeep Garg, Franklin Watkins, Brian R. Ott, Henry Querfurth, Geoffrey Tremont, Stephen Salloway, Paul Malloy, Stephen Correia, Howard J. Rosen, Bruce L. Miller, David Perry, Jacobo Mintzer, Kenneth Spicer, David Bachman, Elizabether Finger, Stephen Pasternak, Irina Rachinsky, John Rogers, Dick Drost, Nunzio Pomara, Raymundo Hernando, Antero Sarrael, Susan K. Schultz, Laura L. Boles Ponto, Hyungsub Shim, Karen Ekstam Smith, Norman Relkin, Gloria Chaing, Michael Lin, Lisa Ravdin, Amanda Smith, Balebail Ashok Raj, Kristin Fargher

**Affiliations:** 1 Department of Software and Computer Engineering, Ajou University, Suwon, South Korea; 2 Biomedical & Translational Informatics Institute, Geisinger, Danville, USA; 3 Center for Computational Biology and Bioinformatics, Indiana University School of Medicine, Indianapolis, IN USA; 4 Center for Neuroimaging, Department of Radiology and Imaging Sciences, Indiana University School of Medicine, Indianapolis, IN USA; 5 The Huck Institute of the Life Sciences, Pennsylvania State University, University Park, USA; 6 UC San Francisco, San Francisco, CA 94107 USA; 7 UC San Diego, La Jolla, CA 92093 USA; 8 Mayo Clinic, Rochester, MN USA; 9 UC Berkeley, Berkeley, San Francisco USA; 10 University of Pennsylvania, Philadelphia, PA 19104 USA; 11 USC, Los Angeles, CA 90032 USA; 12 UC Davis, Sacramento, CA USA; 13 Brigham and Women’s Hospital/Harvard Medical School, Boston, MA 02215 USA; 14 Indiana University, Bloomington, IN 47405 USA; 15 Washington University St. Louis, St. Loui, MO 63110 USA; 16 Prevent Alzheimer’s Disease 2020, Rockville, MD 20850 USA; 17 Siemens, Erlangen, Germany; 18 Alzheimer’s Association, Chicago, IL 60631 USA; 19 University of Pittsburg, Pittsburgh, PA 15213 USA; 20 Cornell University, Ithaca, NY 14853 USA; 21 Albert Einstein College of Medicine of Yeshiva University, Bronx, NY 10461 USA; 22 AD Drug Discovery Foundation, New York, NY 10019 USA; 23 Acumen Pharmaceuticals, Livermore, CA 94551 USA; 24 Northwestern University, Chicago, IL 60611 USA; 25 National Institute of Mental Health, Bethesda, MD 20892 USA; 26 Brown University, Providence, RI 02912 USA; 27 University of Washington, Seattle, WA 98195 USA; 28 University of London, London, UK; 29 UCLA, Torrance, CA 90509 USA; 30 University of Michigan, Ann Arbor, MI 48109-2800 USA; 31 University of Utah, Salt Lake City, UT 84112 USA; 32 Banner Alzheimer’s Institute, Phoenix, AZ 85006 USA; 33 UUC Irvine, Orange, CA 92868 USA; 34 Johns Hopkins University, Baltimore, MD 21205 USA; 35 Richard Frank Consulting, Consulting, USA; 36 National Institute on Aging, Baltimore, MD USA; 37 Oregon Health and Science University, Portland, OR 97239 USA; 38 University of Alabama, Birmingham, AL USA; 39 Mount Sinai School of Medicine, New York, NY USA; 40 Rush University Medical Center, Chicago, IL 60612 USA; 41 Baylor College of Medicine, Houston, TX USA; 42 Wien Center, Miami Beach, FL 33140 USA; 43 Columbia University Medical Center, New York, NY USA; 44 New York University, New York, NY USA; 45 University of Texas Southwestern Medical School, Galveston, TX 77555 USA; 46 Duke University Medical Center, Durham, NC USA; 47 Emory University, Atlanta, GA 30307 USA; 48 University of Kansas Medical Center, Kansas City, KS USA; 49 University of Kentucky, Lexington, KY USA; 50 Mayo Clinic, Jacksonville, FL USA; 51 University of Rochester Medical Center, Rochester, NY 14642 USA; 52 Yale University School of Medicine, New Haven, CT USA; 53 McGill Univ. Montreal-Jewish General Hospital, Montreal, PQ H3A 2A7 Canada; 54 Sunnybrook Health Sciences, Toronto, ON Canada; 55 U.B.C. Clinic for AD & Related Disorders, Vancouver, BC Canada; 56 Cognitive Neurology - St. Joseph’s, London, ON Canada; 57 Cleveland Clinic Lou Ruvo Center for Brain Health, Las Vegas, NV 89106 USA; 58 Premiere Research Inst (Palm Beach Neurology), W Palm Beach, FL USA; 59 Georgetown University Medical Center, Washington, DC 20007 USA; 60 Stanford University, Stanford, CA 94305 USA; 61 Boston University, Boston, MA USA; 62 Howard University, Washington, DC 20059 USA; 63 Case Western Reserve University, Cleveland, OH 44106 USA; 64 Neurological Care of CNY, Liverpool, NY 13088 USA; 65 St. Joseph’s Health Care, London, ON N6A 4H1 Canada; 66 Dent Neurologic Institute, Amherst, NY 14226 USA; 67 Ohio State University, Columbus, OH 43210 USA; 68 Albany Medical College, Albany, NY 12208 USA; 69 Hartford Hospital Olin Neuropsychiatry Research Center, Hartford, CT 06114 USA; 70 Dartmouth-Hitchcock Medical Center, Lebanon, NH USA; 71 Wake Forest University Health Sciences, Winston-Salem, NC USA; 72 Medical University South Carolina, Charleston, SC 29425 USA; 73 Nathan Kline Institute, Orangeburg, NY USA; 74 University of Iowa College of Medicine, Iowa City, IA 52242 USA; 75 University of South Florida: USF Health Byrd Alzheimer’s Institute, Tampa, FL 33613 USA

Correction to: *Scientific Reports* 10.1038/s41598-018-37769-z, published online 13 February 2019

This Article contains errors. A Supplementary Information file was omitted from the original version of this Article.

The Supplementary Information file is now linked to this correction notice.

## Supplementary Information

Supplementary Information.

