# Supplementary material for: Author Correction: Predicting Alzheimer’s disease progression using multi-modal deep learning approach
Source: Sci Rep. 2023 Aug 1;13:12466. doi: 10.1038/s41598-023-39138-x (PMC10393953; doi:10.1038/s41598-023-39138-x)
Supplement: Supplementary file 1 — Supplementary Information. [file 41598_2023_39138_MOESM1_ESM.docx]

**Gated Recurrent Unit**

GRU is an extension of the RNN in which additional parameters regulate the memory state, making it possible to “forget” irrelevant, outdated past information. GRU introduces 2 gates: the update gate $z$ and the reset gate $r$ (Supplementary Figure 1). The update gate $z$ selects whether the memory state $h_{t}$ is to be updated (Eqn. 4), and the reset gate $r$ decides whether the previous memory state is ignored (Eqn. 3). For example, if the reset gate is set to zero, the previous memory state $h_{t-1}$ is not considered to compute the current output, and thus the next memory state is less related to $h_{t-1}$. If the update gate is set to zero, then the next memory state will not be updated, retaining its previous value $h_{t-1}.$ Those units that learn to capture short-term dependencies will tend to have reset gates that are frequently active, but those that capture longer-term dependencies will have update gates that are mostly active.

|  | $z=\sigma\left( x_{t}U_{z}+h_{t-1}W_{z} \right)$ | (1) |
| --- | --- | --- |
|  | $r=\sigma\left( x_{t}U_{r}+h_{t-1}W_{r} \right)$ | (2) |
|  | $y_{t}=tanh\left( x_{t}U_{y}+\left( h_{t-1}\circ r \right)W_{y} \right)$ | (3) |
|  | $h_{t}=\left( 1-z \right)\circ y_{t}+z\circ h_{t-1}$ | (4) |

|  | 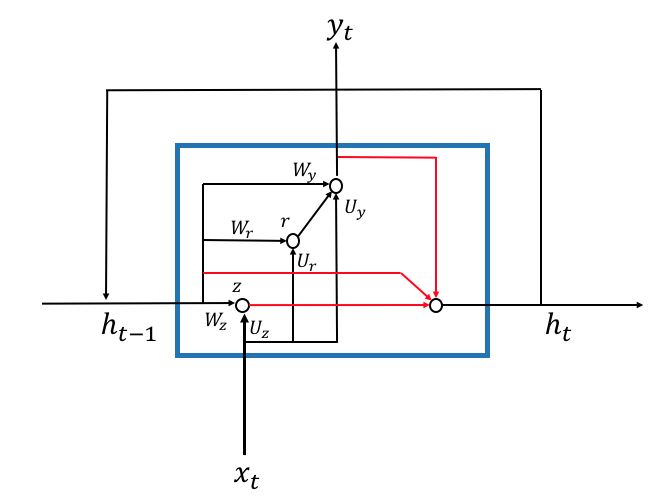 |  |
| --- | --- | --- |
|  | **Supplementary Figure 1.** Gated Recurrent Unit has additional vectors: update gate vector and reset gate vector. $U$ and $W$ are input-specific and memory state-specific weight matrix, respectively. Arrows in memory state (blue rectangle) represents information flows to compute gate vectors and outputs. Points for computing memory state $h_{t}$ are colored in red for visual convenience. |  |
